# A review of foodborne *Toxoplasma gondii* with a special focus on its prevalence in Pakistan from 2000 to 2022

**DOI:** 10.3389/fvets.2022.1080139

**Published:** 2023-01-18

**Authors:** Warda Qamar, Abdullah F. Alsayeqh

**Affiliations:** ^1^Department of Parasitology, University of Agriculture, Faisalabad, Pakistan; ^2^Department of Veterinary Medicine, College of Agriculture and Veterinary Medicine, Qassim University, Buraidah, Saudi Arabia

**Keywords:** *Toxoplasma*, life cycle, transmission, symptoms, prevalence, diagnostic methods/prevention

## Abstract

Third-world countries have a higher prevalence of food-related disorders than developed nations. Millions of people in underdeveloped countries are seriously at risk from the potential water supply contamination with protozoan diseases. *Toxoplasma gondii* is one of the important protozoans causing diseases in livestock and humans. Despite the standard tests for diagnosing this parasite and different treatment methods, the spread of these parasites is uncontrollable and rising every year due to other management disorders. In this review, we summarize etiopathogenesis and prevalence in Pakistan. We looked for papers reporting the seroprevalence of *T. gondii* in people and animals between 2000 and 2022 in different databases: PubMed, Google Scholar, ScienceDirect, Scopus, and Web of Science. Data on the seroprevalence of *T. gondi*i in Pakistan's domestic animals (sheep and goats, horses, donkeys, mules, cattle, and buffaloes), domestic pets (cats and dogs), poultry and rodents, and humans were gathered. According to the findings, sheep had an estimated pooled seroprevalence of *T. gondii* that varied from 11.20 to 26.50 %, and goats from 24.50 to 38.40%. Whereas in buffalo the opposite trend was followed, and the prevalence was observed is 0% in 2022, in horses, donkeys, and mules, only one study was reported according to which a high prevalence was observed in mules (28.60%) followed by donkeys (23.50%) and horses (23.50%), in cats 38.5% prevalence was observed in a recent study and in dogs 28.43% observed, and in humans from 22 to 60%. Human beings are found to be the most affected species showing high prevalence among all. According to our findings, animals and pets not only serve as a reservoir for the parasite but also serve as a direct route for human infection with *T. gondii*. The diagnostic techniques used in the observed studies were mostly serological testing whereas only a few studies have only been observed with molecular testing. To know the exact pattern of the disease for its control, the trend of molecular and advanced testing should be adopted as it is more reliable. Moreover, to decrease the transmission chances of *T. gondii* to humans, it is crucial to manage *T. gondii* infections in non-human species.

## 1. Introduction

Infections caused by food and water have attracted a lot of attention recently. The term “foodborne sickness” refers to a set of diseases that develop after consuming microbially or chemically contaminated food. Even contaminated water, utensils, as well as the hands of the diner can spread the disease. Third-world countries have a higher prevalence of food-related disorders than developed nations. Most people in the world still lack access to clean water and sanitary facilities, and households in rural areas where untreated water used for drinking, cooking, washing fruits, bathing, and swimming expose them to various pathogens including protozoan parasites (1, 2). Millions of people in underdeveloped countries are seriously at risk from the potential water supply contamination with protozoan diseases. There are many basic signs of food-related diseases, and gastrointestinal dysfunction is commonly used to diagnose them.

Parasites are capable of causing an acute, chronic, and debilitating type of diseases (3–6). In nature, parasitic protozoa may be found almost everywhere. In both developed and developing nations, they are accountable for epidemics and chronic poverty (7). Since certain parasites are zoonotic in nature and consequently exist in animals, their food and water prevalence should be considered a public health problem (8). The prevalence of food- and waterborne parasites has increased throughout time due to several past disease outbreaks linked to parasites. The World Health Organization (WHO) and the Food and Agriculture Organization of the United Nations (FAO) published their worldwide risk rating of foodborne parasites (FBPs) in 2014 (2). It was followed in 2015 as a global burden related to foodborne pathogens (9). Despite being acknowledged as significant foodborne pathogens, parasites are still underappreciated when compared to bacterial and viral foodborne pathogens (10).

*Toxoplasma* spp is one of the important protozoans causing disease in livestock and humans (11–13). Across the world, this parasite has posed a serious threat. Despite the standard test for diagnosing this parasite and different treatment methods, the spread of these parasites is uncontrollable due to the other management disorders (14). This review summarizes etiopathogenesis, epidemiology in Pakistan from 2000 to 2022, and preventive measures for zoonotic toxoplasmosis.

## 2. Material and methods

### 2.1. Search technique

We searched databases (PubMed, Embase, Google Scholar, ScienceDirect, Scopus, ProQuest, and Web of Science) for articles reporting the seroprevalence of *T. gondii* in Pakistan from 2000 to 2022 to conduct this systematic review. The searches were limited to English-language articles. Electronic searches mostly employed the MeSH keywords (Human, Animal) AND (*Toxoplasma gondii* OR toxoplasmosis; Prevalence and seroprevalence OR serology).

For management, the citations were observed keenly. The final article choice was made after screening each article's title and abstract by eliminating duplicate records.

### 2.2. Criteria for inclusion and exclusion

**Figure 1:**
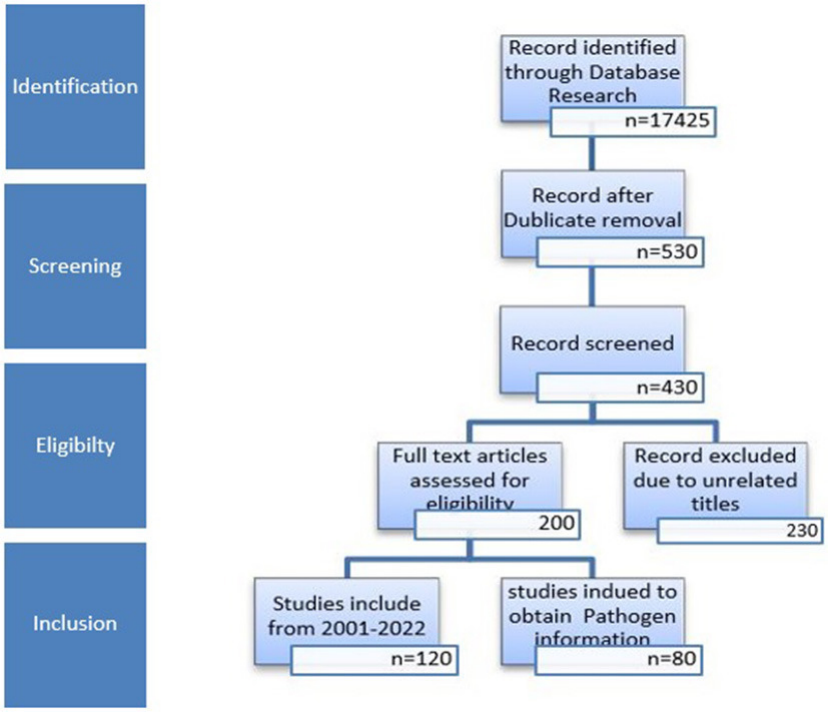
Overview of the number of studies from identification to inclusion.

Using the titles as a guide, references were screened, and unnecessary and duplicate references were removed. A flow chart of the article identification, screening, eligibility, and inclusion criteria is shown in Figure 1. The last search was conducted on October 6th, 2022.

## 3. Toxoplasmosis

*Toxoplasma gondii* is a member of the phylum Apicomplexa, which is made up of a variety of protists, most of which are intracellular parasites capable of inflicting potentially life-threatening diseases in both humans and animals. Given its ability to infect virtually all warm-blooded vertebrates, *T. gondii* is the most prevalent. It is also thought to infect about one-third of the world's population of humans (15). According to the nation or region under consideration, seropositivity rates in the human population vary from < 10 to over 90%, partly due to local socio-economic conditions and population patterns (16). For instance, there is a more significant incidence in continental Europe, South America, and the United Kingdom than in the United States or the United Kingdom. Both wild and domestic animals have high seroprevalence, making them essential *T. gondii* reservoirs and sources for human contamination through meat intake (17). In addition to being an issue for human contamination, toxoplasmosis in farm animals has a significant negative impact on the livestock (on milk production and reproductive performance), which results in a high cost for the industry (18).

Only one species has been identified for the genus *Toxoplasma*, yet many clonal lineages have varying degrees of pathogenicity. Four primary clonal types I, II, III, and XII dominate the population pattern of *T. gondii* in Europe and North America (19–21). The most common strains in a wild and domestic context in Europe are type II (and type III, though to a lesser extent) (22, 23). Domestic isolates from North America are comparable to those from Europe (types II and III), while in the wild, strains from type XII prevail (24, 25). More contrast exists in other regions of the world. For instance, South America has a lot more genetic variety (26, 27), which suggests that recombination occurs more frequently there. Following *T. gondii* infection, the host's type, genetic makeup, and of course, the host's immune status all play a role in the development of the disease. Some species appear to be innately resistant to *T. gondii* infection. In contrast, others are highly susceptible, partly due to variables like their habitat's closeness to the parasite's definitive hosts (28). However, the host immune system and how parasite factors affect it continue to be one of the most critical factors affecting susceptibility to *T. gondii* (29–32).

## 4. Life cycle and routes of transmission of *Toxoplasma*

**Figure 2:**
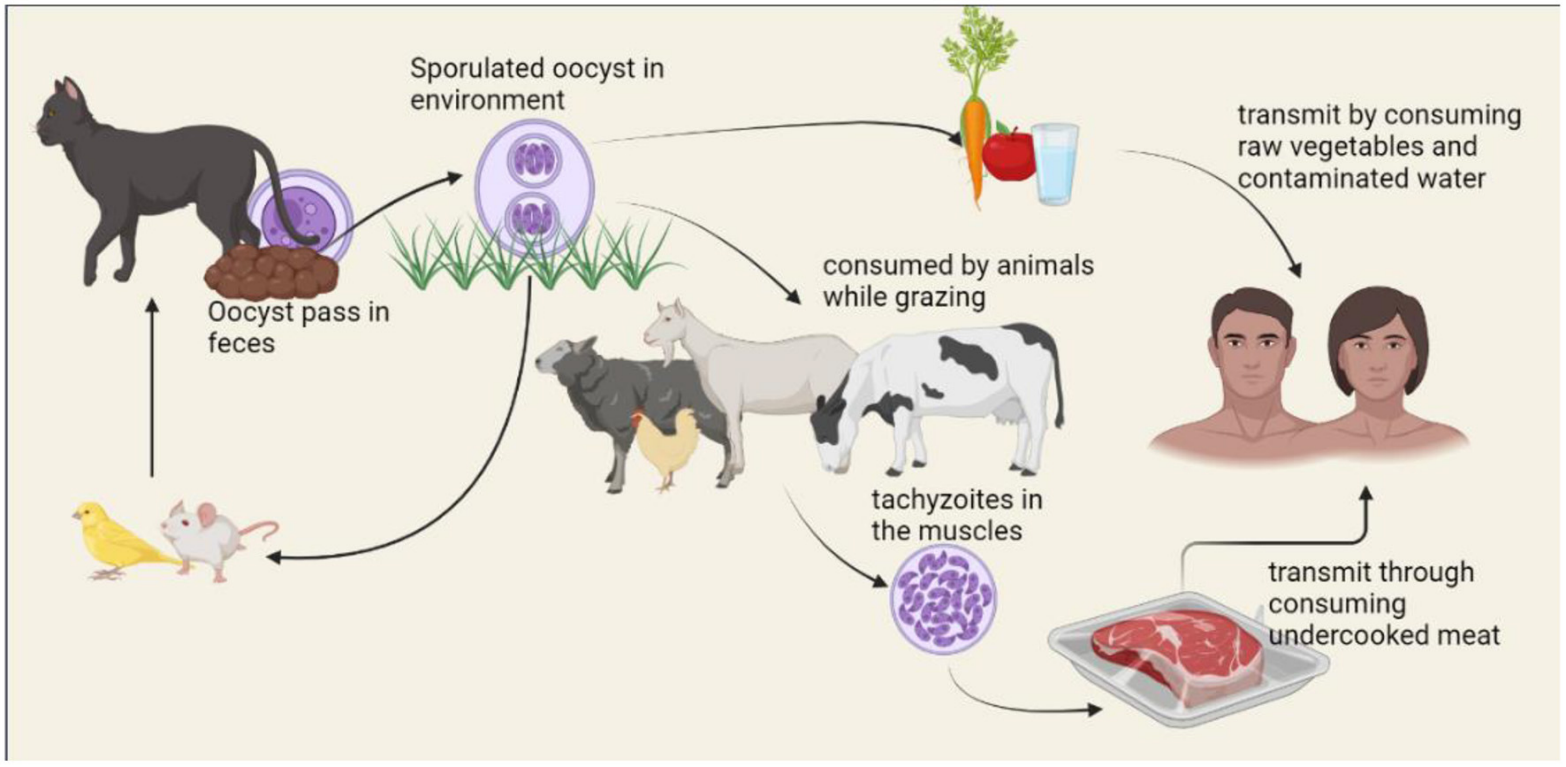
Life cycle of *Toxoplasma gondii*.

The life cycle of *T. gondii* includes both, asexual replication in a range of vertebrate hosts (intermediate hosts) and sexual replication in felids (definitive hosts). Felids consume the *T. gondii* by consuming encysted bradyzoites on infected intermediate hosts. Under the influence of digestive enzymes and acid, bradyzoites are liberated from cysts and enter the small intestine's epithelial cells. Although the parasite may spread throughout the body of the final host and cause clinical symptoms, this is uncommon (33). More typically, bradyzoites transform into schizonts in the intestine before reaching the merozoite stage (34). Merozoites differentiate into male and female gametes after a few cycles of asexual division. After that, male and female gametes combine to form diploid oocysts, which are enclosed in a solid, impenetrable wall. The millions of them contaminate the ecosystem that the felids excrete. The oocysts are resilient and survive in the environment, allowing them to spread (35, 36). Intermediate hosts consume sporulated oocysts by drinking or eating contaminated water and foods. Invading sporozoites quickly transform into the tachyzoite form inside a transitory parasitophorous vacuole (PV) that stays in the host cells (37). Tachyzoites are proliferative forms of toxoplasmosis that spread throughout the body and cause acute symptoms. They can move between tissues *via* blood vessels or the lymphatic system. At least when felids can prey on the intermediate host, this ensures parasite transmission to the final host to finish the cycle. Even when intermediate hosts aren't the felids who are often their prey, the parasites can still spread to new intermediate hosts through carnivory, keeping the parasite transmission cycle going without the necessity for sexual reproduction. The life cycle of *T. gondii* is shown in Figure 2.

Human infection can occur through food consumption, such as raw or undercooked meat containing cysts or vegetables, fruits, or water that has sporulated oocysts (38). To prevent foodborne toxoplasmosis, it is crucial to wash produce, cook meat properly, and adequately treat sewage or water (39). Congenital transfer of tachyzoites from a woman who is mainly infected to the growing fetus through the placenta is one of the alternative ways of transmission (40).

Congenital toxoplasmosis must be managed with precautions that restrict the mother's exposure to established transmission channels while pregnant and with quick identification and treatment beginning following infection. Although uncommon, blood transfusion (41) or organ transplantation (42) from sick donors are potential sources of contamination in people.

## 5. Clinical manifestations of toxoplasmosis

In immunocompetent people, toxoplasmosis can cause a minor, self-limiting disease or remain unnoticed in most cases (43). In pregnant women, congenital toxoplasmosis may develop because, during the parasite's dissemination phase, the parasite passes through the placenta and infect the growing fetus. Depending on the gestational stage at the time of maternal infection, it might result in varying degrees of neurological, ophthalmic, or systemic damage. For instance, a maternal illness in the first trimester may result in more severe symptoms (44). Although some of them can also happen later in life, hydrocephalus, mental retardation, epilepsy, and blindness are the most significant sequelae for newborns (45).

Even though acute acquired infection can occur, immunodeficiency in adults can also result in severe toxoplasmosis.

People who have impaired immune systems or immunosuppression [such as those with HIV (46), cancer patients (47), or transplant recipients] are particularly vulnerable. Toxoplasmic encephalitis may be the most severe outcome because it causes significant tissue damage and inflammation when toxoplasmosis from parasites ensconced in the central nervous system returns (48). Left untreated, this cerebral toxoplasmosis can be potentially fatal and frequently manifest as headache, fever, ataxia, or seizures. Acute toxoplasmosis has harmful effects, but chronic toxoplasmosis—the parasite's long-term persistence in the body as tissue cysts—may also have significant effects on behavioral changes and psychiatric problems, especially because it affects the central nervous system (49).

## 6. Prevalence of toxoplasmosis in humans and animals of Pakistan

Human studies that assessed the seroprevalence of *T. gondii* among the various individual groups listed in Table 1 have been published in Pakistan. Seroprevalence of *T. gondii* in dogs and cats, small ruminants, large ruminants, equines and camels, and poultry are mentioned in Tables 2–6, respectively. Pregnant women's seroprevalence has received the majority of attention in research (50–52), followed by patients with illnesses (53), The sociodemographic information, epidemiological profile, potential risk factors for transmitting the *T. gondii* infection, and the source of detection and diagnostic approach is also the focus of these investigations. Variable seroprevalence levels have been observed with a rise in percentage between 2001 and 2022.

**Table 1:** Reported prevalence of *Toxoplasma gondii* in human population of Pakistan.

**Species**	**Area of study**	**Prevalence rate (%)**	**Source of detection**	**No. of samples tested**	**Diagnostic approach**	**Age of animal**	**Year of study**	**References**
Human	Lahore	22%	Serum	150	LAT	< 15 years to >40 years	N/A	(59)
	Kohat, Khyber-Pakhtunkhwa	14.4%	Serum	180 pregnant	ELISA	N/A	June–September 2007	(60)
	Lahore	11.33%	Serum	300	LAT	Not mentioned for rats ≥45 years in humans	2012	(61)
	Rajanpur, Bahawalnagar, and Multan	29.45%	Serum	550	LAT	1–70 years	2010	(62)
	Khyber-Pakhtunkhwa	65.71%	Serum	420	ELISA	10 to >51 years	N/A	(63)
	Multan	19.4% pregnant women 15.2% non-pregnant women	Serum	232 pregnant women 171 non-pregnant women	ELISA	20–40 years	2017	(64)
	Khyber-Pakhtunkhwa	1.32%	Serum	150 pregnant women	ELISA	20–40 years	February–November 2015	(65)
	Charsadda	21%	Serum	300	LAT	15–75 years	May–July 2017	(66)
	Sub-Tropical Areas	20.37%	Serum	1,659	ELISA	< 10 to >40 months	N/A	(67)
	Punjab	7.42%	Serum	593	ELISA	< 20 to >40 years	January 1–December 31, 2017	(68)
	Bahawalpur	21.2% farmer 6.8% non-farmer	Serum	160 farmer 160 non-farmer	LAT	N/A	May 2016–April 2017	(69)
	Swat	25.92%	Serum	216	Lateral flow chromatographic immune-assay	31–40 years	June–September 2016	(70)
	Khyber-Pakhtunkhwa	40.6%	Serum	360	ELISA	16–40 years	N/A	(53)
	Peshawar	21.3%	Serum	94 Pregnant women	ICT	21–53 years	September–December 2017	(52)
	Sahiwal	24.5%	Serum	200	ELISA	N/A	May–November 2020	(71)
	KPK	39.94%	Serum	425	ELISA	15–50 years	N/A	(72)
	Khanewal	52%	Serum	200	ELISA	N/A	May–November 2020	(71)

**Table 2:** Reported prevalence of *Toxoplasma gondii* in cats and dogs in Pakistan.

**Species**	**Area of study**	**Prevalence rate (%)**	**Source of detection**	**No. of samples tested**	**Diagnostic approach**	**Age of animal**	**Year of study**	**References**
Cats	Faisalabad	60%	Serum	10	LAT	6 months to >4 years	N/A	(73)
	Lahore	56% cats	Serum	50	LAT	6 months	N/A	(59)
	Sub-tropical Arid parts	26.43% cats	Serum	420	ELISA	1–2 years	January–December 2012	(74)
	Lahore	2.3%	Feces	470	PCR	N/A	June 2013–May 2014	(75)
	KPK	25.4% cats	Serum and blood	50	PCR	2–4 years	N/A	(76)
	KPK	74.6%	Serum and blood	147	ELISA	2–4 years	N/A	(76)
	KPK	2.50%	Feces	40	Centrifugal sedimentation leading to PCR	N/A	January–December 2019	(77)
	Sahiwal and Khanewal	6.5%	Feces	200	Floatation/sedimentation	N/A	May–November 2020	(71)
	KPK	12.22%	Serum	40	LAT	N/A	January–December 2019	(77)
	Sahiwal and Khanewal	38.46%	Feces	13	PCR	N/A	May–November 2020	(71)
Dogs	Faisalabad	50% dogs	Serum	40	LAT	6 months to >4 years	N/A	(73)
	Lahore	39% dogs	Serum	100	LAT	6 months to >7 years	N/A	(59)
	Lahore	46.88%	Serum	305	LAT	6 months to >4 years	N/A	(78)
	Sub-tropical Arid parts	28.43% Dogs	Serum	408	ELISA	1–2 years	January–December 2012	(74)

**Table 3:** Reported prevalence of *Toxoplasma gondii* in small ruminants (sheep and goats) in Pakistan.

**Species**	**Area of study**	**Prevalence rate (%)**	**Source of detection**	**No. of samples tested**	**Diagnostic approach**	**Age of animal**	**Year of study**	**References**
Sheep	Rahim Yar Khan	11.2%	Serum	90	LAT	N/A	2006–2007	(79)
	Mardan	44.13% sheep	Serum	290	IHA	1–2 years	N/A	(80)
	Pothwar Region	18.16% sheep	Serum	413	ELISA	1–3 years	September 2011–December 2012	(81)
	Southern Punjab	37.31%	Serum	335	LAT	N/A	May 2012–April 2013	(82)
	Northeast Punjab	26.2% sheep	Serum	470	ELISA	1–3 years	January–December 2013	(83)
	Multan	34.02%	Serum	288	LAT	4–73 months	April 2012–June 2013	(84)
	Khanewal	33.01%	Serum	212	LAT	4–73 months	April 2012–June 2013	(84)
	Cholistan desert (Punjab)	37.31%	Serum	335	LAT	1 to >25 months	N/A	(84)
	Cholistan desert (Punjab)	29.13%	Serum	865	LAT	1 to >25 months	N/A	(74)
	Multan	44.80%	Serum	125	LAT	< 1 to > 2 years	N/A	(85)
	Charsadda	40.55% sheep	Serum	143	LAT	1–4 years	N/A	(86)
	Dera Gazi Khan	23% ELISA 25% LAT	Serum	103	ELISA and LAT	8–42 months	N/A	(87)
	Bahawalpur	36.25% sheep	Serum	160	LAT	N/A	May 2016–April 2017	(69)
	Peshawar	49% sheep	Serum	360	IHT	< 1 to < 2 years	N/A	(88)
	Khyber-Pakhtunkhwa	52.69%	Serum	167	ELISA	1 to > 3 years	2018–2020	(54)
	Jhang	31.49% sheep	Serum	181	LAT	< 12 to >24 months	N/A	(90)
	Sahiwal	23.5%	Serum	1,000	ELISA	N/A	May–November 2020	(71)
	Khanewal	26.5%	Serum	1,000	ELISA	N/A	May–November 2020	(71)
**Goats**	Rahim Yar Khan	24.5%	Serum	110	LAT	N/A	2006–2007	(79)
	Mardan	42.28% goats	Serum	350	IHA	1–2 years	N/A	(80)
	Pothwar Region	14.32% goats	Serum	419	ELISA	1–3 years	September 2011–December 2012	(81)
	Northeast Punjab	42.8% goat	Serum	530	ELISA	1–3 years	January–December 2013	(83)
	Multan	40.80	Serum	125	LAT	< 1 to >2 years	N/A	(85)
	Charsadda	41.61%	Serum	149	LAT	1 to >3 years	N/A	(65)
	Bahawalpur	28.1% goat	Serum	160	LAT	N/A	May 2016–April 2017	(69)
	Dera Gazi Khan	32.67% ELISA 35.64% LAT goat	Serum	101	ELISA and LAT	8–42 months	N/A	(87)
	Khyber Pakhtunkhwa	7.9%	Serum	70	ELISA	1 month to < 2 years	2018–2020	(54)
	Dera Ghazi Khan	10%	Serum	410	LAT	1–3 years	6 months	(77)
	Khyber-Pakhtunkhwa	18.25 %	Serum	126	ELISA	1 to >3 years	2018–2020	(54)
	Faisalabad	33.59%	Serum	384	LAT	< 2 to >5 years	October 2016–March 2017	(91)
	Peshawar	45.7% goats	Serum	420	IHA	< 1 to < 2 years	N/A	(88)
	Faisalabad	53.15%	Serum	380	LAT	1–6 years	N/A	(89)
	Khanewal	5.3%	Blood	898	PCR	1–3 years	March 2019–February 2020	(92)
	Faisalabad	17.9%	Serum	240	LAT	< 1 to >3 years	September 2016, February 2017	(93)
	Khanewal	29.2%	Serum	1,000	ELISA	N/A	May–November 2020	(71)
	Jhang	36.52% goat	Serum	219	LAT	< 12 months to >24 months	N/A	(90)
	Sahiwal	38.4%	Serum	1,000	ELISA	N/A	May–November 2020	(71)

**Table 4:** Reported prevalence of *Toxoplasma gondii* in large ruminants (cattle and buffalo) in Pakistan.

**Species**	**Area of study**	**Prevalence rate (%)**	**Source of detection**	**No. of samples tested**	**Diagnostic approach**	**Age of animal**	**Year of study**	**References**
Cattle	Northern Punjab	19.75% cattle	Serum	400	ELISA	>24 to >48 months	January–December 2012	(94)
	Charsadda	55.39%	Serum	139	LAT	1 to >5 years	N/A	(65)
	Khyber Pakhtunkhwa	13%	Serum	100	ELISA	1 month to < 2 years	2018–2020	(54)
	Khyber-Pakhtunkhwa	18 %	Serum	100	ELISA	1 to >3 years	2018–2020	(54)
	Rajanpur	12.2% cattle	Blood	190	PCR	≥5 years to < 5 years	July–October 2019	(95)
	Pakistan	29.75%	Serum	90	ELISA	N/A	N/A	(96)
	Pakistan	35.75%	Serum	400	LAT	>5 years	N/A	(96)
Buffalo	Northern Punjab	15.16% buffalo	Serum	422	ELISA	>24 to >48 months	January–December 2012	(94)
	Charsadda	17.32% Buffalo	Serum	127	LAT	1–4 years	N/A	(86)
	KPK	15.51 %	Serum	58	ELISA	1 to >3 years	2018–2020	(54)
	Rajanpur	0% Buffalo	Blood	120	PCR	≥5 to < 5 years	July–October, 2019	(95)

**Table 5:** Reported prevalence of *Toxoplasma gondii* in equines and camels of Pakistan.

**Species**	**Area of study**	**Prevalence rate (%)**	**Source of detection**	**No. of samples tested**	**Diagnostic approach**	**Age of animal**	**Year of study**	**References**
Equines	Faisalabad, Lahore, and Gujranwala	Horses 23.5%	Serum	183 horses	LAT	≥5 years to < 10 years	N/A	(101)
	Faisalabad, Lahore, and Gujranwala	Mule 28.6%	Serum	14 mules	LAT	≥5 years to < 10 years	N/A	(101)
	Faisalabad, Lahore, and Gujranwala	Donkey 58.7%	Serum	75 donkey	LAT	≥5 years to < 10 years	N/A	(101)
Camels	Bahawalpur Region	10%	Serum	100 camels	LAT	1–15 years	N/A	(102)
	Bhawalpur, Punjab	17.9%	Serum	201 camel	LAT	1–>13 years	February–December, 2015	(103)
	Punjab	40.1%	Serum	897 one-humped camel	Indirect ELISA	3–7 years	July–August, 2016	(104)
	Mianwali district	38%	Serum	350 camels	Indirect ELISA	3–7 years	N/A	(105)

**Table 6:** Reported prevalence of *Toxoplasma gondii* in poultry and rats in Pakistan.

**Species**	**Area of study**	**Prevalence rate (%)**	**Source of detection**	**No. of samples tested**	**Diagnostic approach**	**Age of animal**	**Year of study**	**References**
Poultry	Mardan	18.85%	Serum	536	IHA	N/A	N/A	(96)
	Faisalabad	36.33%	Serum	300	LAT	< 1 to >2 years	July 2011–June 2012	(97)
	Kasur	12.5 %	Serum	200 wild birds	LAT	N/A	N/A	(98)
	Upper dir and Peshawar	10.84	295 tissue samples	295	PCR	30 days to 2 years	N/A	(99)
	Upper Dir and Peshawar	26.6%	398 serum	398	ELISA	30 days to 2 years	N/A	(99)
	Punjab	38.3%	Brain sample	120 rock birds	PCR	N/A	July 2018–October 2018	(100)
Rats	Lahore	58.57% of rats	Serum	Rat 210	LAT	N/A	2012	(61)

IFA, indirect fluorescent assay; IHA, indirect hemagglutination test; ELISA, enzyme-linked immunosorbent assay; LAT, latex agglutination test; PCR, polymerase chain reaction.

The procedure used in the publications is often the collection of a blood sample from the population and testing it for anti-*toxoplasma* antibodies in the sera. The ELISA, latex agglutination (LA) test, and other serological assays were often employed (54, 55). Although their sensitivity and specificity varied, commercial test kits were employed, and the results were occasionally inconclusive.

In the food chain, which serves as a source of nutrition for humans and other animals, animals play a crucial role. The bradyzoite cyst is present in the body tissues of animals, and the parasite then transmits to new hosts by eating a raw or undercooked piece of the infected tissues (56). This situation raises the risk of zoonotic infection by foodborne pathogens since a particular group of humans, such as hunters, butchers, and consumers may get infected by ingesting domestic or wild meat (57). Only wild and domestic cats excrete the oocyst infective stage, which may infect humans when eaten in tainted food, water, or vegetables, which is especially noteworthy (58).

**Figure 3:**
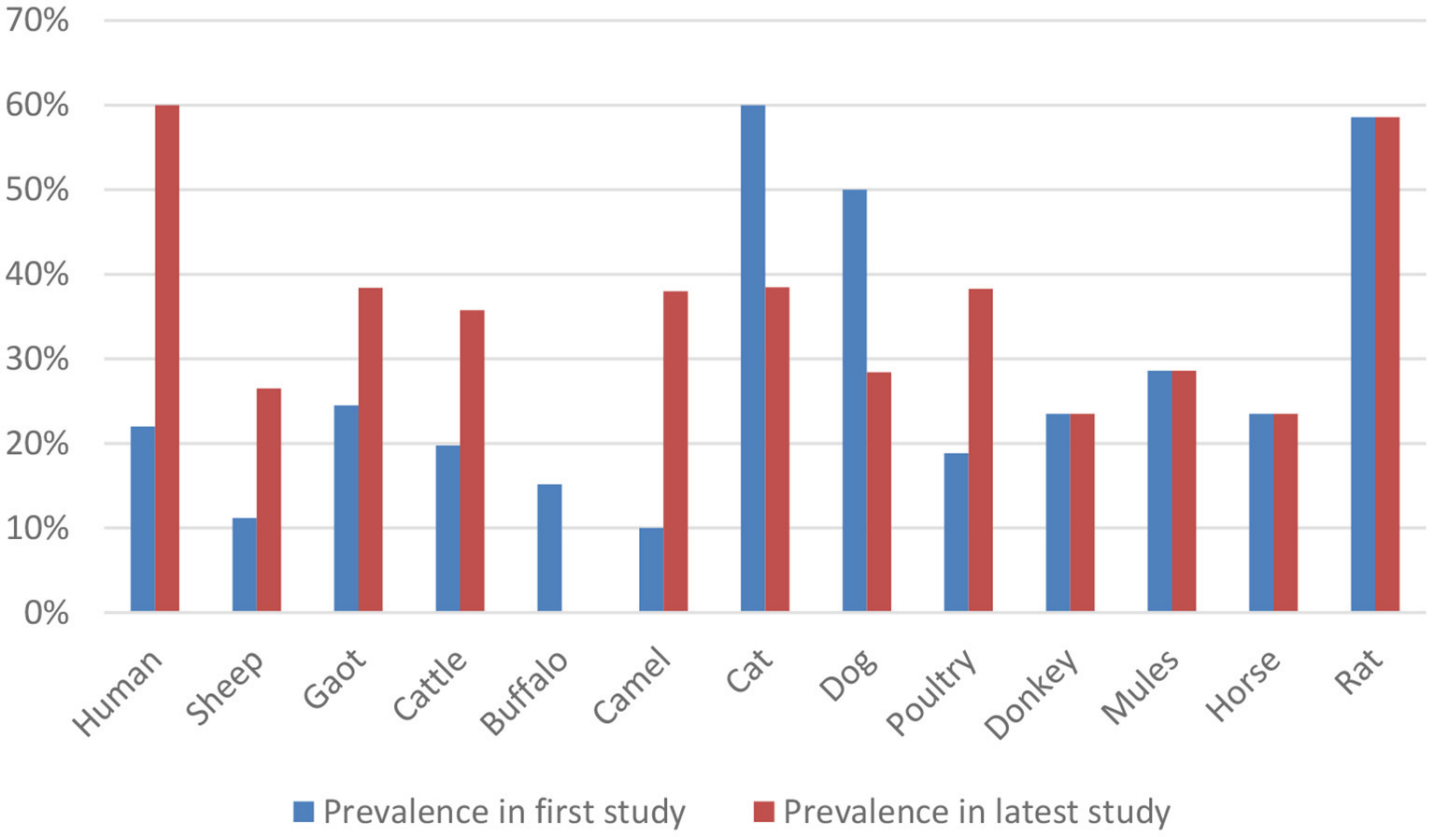
Comparison of prevalence rate of *Toxoplasma gondii* between the first and the latest study.

According to an analysis of the records currently available over the previous 20 years (2001–2022), domestic animals in Pakistan have a relatively high and rising seroprevalence rate of *T. gondii* as a zoonotic infection, except for buffalo, cats, and dogs. The comparison is shown in Figure 3. Although the prevalence level may have decreased and increased in different species, caution is advised.

All the data mentioned in the previous study of Figure 1 is collected from the year 2022 except for equines, dogs, and rats because the previous study performed in equine was in the year 2015, and for rat, it was in 2012. Whereas in dogs, the last study was performed in 2014 in Pakistan.

## 7. Methods used for the detection of *T. gondii*

Examining the levels of immunoglobulin G (IgG), immunoglobulin M (IgM), and IgG avidity in a sample—typically serum from the blood of a particular host population—the serological test assesses the antibodies and calculates the seroprevalence of infection. Although it is the simplest and most straightforward test, it frequently yields false-positive or false-negative findings (106). Most of the studies in Pakistan have been diagnosed through Latex Agglutination Test (LAT). Cd4 mentioned in Table 1. The tests using molecular methods are reliable, perceptive, and accurate (107). Few studies have been reported and mentioned in Table 1, which have been diagnosed with Molecular methods. They use a variety of samples to find a specific gene of interest that is unique to this particular organism. Numerous techniques are routinely used, including loop-mediated amplification (LAMP), quantitative PCR, and traditional polymerase chain reaction (PCR) (108). This technique is seldom employed in histological procedures. It is primarily concerned with identifying the bradyzoite stage in. tissues such as the heart, liver, and brain. The bradyzoite stage is primarily detected in tissues, including the heart, liver, and brain (109). Before being examined under a microscope, such tissues are mounted on a glass slide and stained with hematoxylin and eosin (H&E). Another method of evaluating suspected samples, such as cat feces, liver, lung, and brain homogenates of intermediate hosts by inoculation and then testing the animal for the presence of an infection, is bioassay/*in vivo* using an animal model (mice/rat) (110). The test is costly and time-consuming, but it is an accurate approach to assessing the sustainability and pathogenicity of the various strains. Through the establishment of an enclosed environment where suspected specimens, such as blood, are cultured in a medium, the *in vitro*/tissue culture technique removes the usage of animals (111). Microscopy is used to assess the sample's motility or viability for the tissue culture endpoint. Most intuitive findings that can identify the parasite's morphology nevertheless rely heavily on microscopy as their foundation. Other tests like tissue culture and histology consistently rely on it because of its adaptability (112).

## 8. Comparison of serological techniques for *T. gondii* antibody detection in Pakistan

All studies employed convenient sampling to gather data, and two serological tests—the Latex Agglutination Test (LAT) and Enzyme-Linked Immunosorbent Assay (ELISA)—are mostly used to assess the outcomes based on the detection of IgG, IgM, and avidity test of T. *gondii* antibodies (Table 1) (96, 99). Most studies did not follow established procedures for collecting and processing specimens, and most did not have information on the control group. However, since a different company produced each ELISA and LAT test, it was challenging to evaluate and confirm each assay's specificities and sensitivities. Except for a few recent studies, further PCR validation of the data was not done (75, 77, 95, 100). Because of differences in the experimental design and the commercial kits utilized, some of the results are thus disputed.

## 9. Prevention of toxoplasmosis

The foundation for the current strategies employed to control *T. gondii* infection has been supplied by the exponential growth in our understanding of *T. gondii* biology, epidemiology, and ecology during the past few decades. Limiting contact with available transmission channels and minimizing exposure to the parasite's infectious phases are the main goals of preventative interventions.

As previously indicated, people contract *T. gondii* either by eating or drinking raw or undercooked meat with parasite cysts on it or by drinking water contaminated with oocysts deposited in cat feces. Additionally, eating raw shellfish can result in illness (106).

Therefore, seronegative should only consume fully cooked meat, refrain from consuming raw shellfish, carefully wash their hands after coming into contact with raw meat, avoid gardening and soil handling without gloves, and thoroughly clean fruits and vegetables.

People who take care of the litter box should make it a habit to wear disposable gloves and wash their hands thoroughly with antiseptic. Seronegative should refrain from adopting or handling stray cats, and cats should stay indoors whenever feasible. They should also not be fed raw or undercooked meat. The suggestions mentioned above for preventing *T. gondii* infection also apply to people in other particular at-risk groups. Soon after HIV diagnosis, standardized guidelines advise testing all for serological signs of prior *T. gondii* infection (107).

Primary prophylaxis should be given to those who are also seropositive for *T. gondii* and have peripheral blood CD4 T cell levels of 100/L (107). People receiving cART who have had more than 200 CD4 T cells/L for 3 months can stop using primary prophylaxis without risk. When they reach more than 200 CD4 T cells/L for 6 months, PLWH who have undergone effective therapy for TE and are getting cART can stop receiving maintenance treatment (107). It is important to remember that despite these precautions, *T. gondii* infection cannot be entirely avoided.

## 10. Conclusion

Infectious diseases of animals including parasitic infestations pose significant threats to health and productivity potential of animals (113–116) which leads to heavy economic losses (117–122). Parasitic infections lead to chronic and debilitating types of diseases and have zoonotic implications as well (3, 123–126). Results of the current evaluation on toxoplasmosis research in Pakistan from 2001 to 2022 revealed little information on animal seroprevalence in cases of humans, cattle, buffaloes, sheep, goats, cats, dogs, camels, and horses. In Pakistan, the seroprevalence among human females is rising. The frequency of toxoplasmosis in cattle, mainly chicken intended for human consumption, is also little understood. The *T. gondii* strain prevalent in Pakistan from HIV patients, pregnant women, livestock, and domestic cats has not yet been genetically characterized. Alarming reports of toxoplasmosis in the KPK population have been observed in humans. Despite the widespread occurrence and severe effects of toxoplasmosis, which are mostly seen in immunocompromised patients, there are significant flaws in the present control programs, particularly in the diagnostic resources available. The prevalence throughout the country is increasing every year. The majority of diagnostic procedures also frequently misdiagnose the illness in endemic regions. It is necessary to create molecular approaches that are sensitive, specific, straightforward to use, affordable, and high throughput because early detection is the most effective way to combat the illness. Researchers, healthcare professionals, veterinary professionals, and politicians can benefit from the current review on toxoplasmosis. Therefore, there is an urgent need to inform and educate the public about the risk factors for toxoplasmosis infection in humans and animals. That may be accomplished by running health-related advertisements and educational campaigns in regional newspapers, television, radio, and, more recently, social media platforms.

## Data availability statement

All data supporting the conclusions of this article are included within the article.

## Author contributions

WQ and AA worked on the development of this unique title of review, planned, designed, and structured the layout of the article. WQ wrote the article. AA reviewed the article. All authors finally approved this review article.
